# Protective Effects of Proanthocyanidin on Cerulein-induced Acute Pancreatic Inflammation in Rats

**DOI:** 10.4021/gr2009.02.1276

**Published:** 2009-01-20

**Authors:** Cebrail Akyuz, Ahmet Ozer Sehirli, Umit Topaloglu, Ayliz Velioglu Ogunc, Sule Cetinel, Goksel Sener

**Affiliations:** a Department of 5^th^ Surgery, Haydarpasa Numune Education and Research Hospital; b Marmara University, School of Pharmacy, Department of Pharmacology; c Marmara University, Vocational School of Health Related Professions; d Marmara University, School of Medicine, Department of Histology & Embryology, Istanbul, Turkey

**Keywords:** Pancreatitis, Proanthocyanidine, Glutathione, Cytokines, Myeloperoxidase activity, Tumor Necrosis Factor alpha

## Abstract

**Background:**

The aim of this study was to assess the possible protective effect of proanthocyanidin against cerulein-induced acute pancreatic inflammation (AP) and oxidative injury.

**Methods:**

Sprague-Dawley rats were pretreated with proanthocyanidine (100 mg/kg, orally) or saline 15 min before cerulein was given by 20 µg/kg subcutaneously at 1-h intervals within 4 hours. Six hours after cerulein or saline injections, the animals were killed by decapitation. Blood samples were collected to analyze amylase, lipase, and proinflammatory cytokines (TNF-α and IL-1b). Pancreas tissues were taken for the determination of tissue glutathione (GSH) and malondialdehyde (MDA) levels, Na^+^, K^+^-ATPase and myeloperoxidase (MPO) activities. Formation of reactive oxygen species in pancreatic tissue samples was monitored by using chemiluminescence (CL) technique with luminol and lucigenin probes, while the extent of tissue injury was analyzed microscopically.

**Results:**

Acute pancreatitis caused a significant decrease in tissue GSH level and Na^+^, K^+^-ATPase activity, which was accompanied with significant increases in the pancreatic MDA, luminol and lucigenin chemiluminescences (CL) levels and MPO activity. Similarly TNF-α and IL-1β levels were elevated in the pancreatic group as compared to control group. On the other hand, proanthocyanidin treatment reversed all these biochemical indices, as well as histopathological alterations that were induced by cerulein.

**Conclusions:**

Proanthocyanidine can ameliorate pancreatic injury induced by cerulein in rats, this result suggests that proanthocyanidin may have utility in treating acute pancreatititis.

## Introduction

Acute pancreatitis (AP) is an inflammatory disease with increasing incidence worldwide. The development of systemic inflammatory response syndrome (SIRS) is one of leading events responsible for the mortality of AP. SIRS results from the excessive release of inflammatory mediators from the local tissues and results in the systemic amplification of AP, which may ultimately cause multiple organ system failure (MOF) within 24-72 hours [1-3]. Many of the systemic features of severe acute pancreatitis can be attributed to the release of proteolytic enzymes, cytotoxic and inflammatory substances, reactive oxygen species, cytokines and other mediators into the circulation and explosive activation of the systemic inflammatory response [4].

Oxidative stress is emerging as the pivotal effector of acinar cell injury in experimental AP, irrespective of the initiating agent or its route of attack [5]. Different oxygen radical species induce severe acinar cell damage in a dose and time dependent manner, which suggests that generation of reactive oxygen species (ROS) may be crucial for initiating the pathophysiologic changes of AP [6].

It is well known that antioxidants are potent scavengers of free radicals and serve as inhibitors of oxidant stress related pathologies. A large number of synthetic and natural antioxidants have been demonstrated to induce beneficial effects on human health and disease prevention. However, the structure-activity relationship, bioavailability and therapeutic efficacy of the antioxidants differ extensively. Proanthocyanidins consist of a group of polyhydroxyl-flavan-3-ol (or flavan-3,4-diol) oligomers and polymers linked by carbon-carbon bonds between flavanol subunits [7]. They are the most abundant natural phenolic components [8, 9], including phenoldienones, epicatechin, epigallocatechin, epigallocatechin gallate, ferulic acid, caffeic acid, *p*-coumaric acid, kaempferol, quercetin, and myricetin derived from common dietary foods such as grapes, cranberries and almonds, as well as chocolate and cacao beans [10, 11]. These compounds have been reported to possess a broad spectrum of biological, pharmacological and therapeutic activities against free radicals and oxidative stress both *in vitro* and *in vivo* [10-13]. Previous studies have shown that proanthocyanidins provide significant protection against free radicals induced lipid peroxidation and DNA fragmentation in liver and brain tissue [13] and provided better protection than vitamin C, vitamin E, and b-carotene [10].

Despite the considerable works done on proanthocyanidin against free radical associated tissue injury, its effect and role in the acute pancreatitis remain to be elucidated. In the present study, therefore, we investigated the protective effect of proanthocyanidin against cerulein-induced pancreatitis and oxidative injury in rats.

## Materials and Methods

### Animals

Sprague–Dawley rats of either sex (200 - 250 g) were kept in a room at a constant temperature 22 ± 1 °C with 12 h light/dark cycles and fed standard pellet chow and water *ad libitum*. All experimental protocols were approved by the Marmara University School of Medicine Animal Care and Use Committee.

### Experimental protocol

Acute pancreatitis was induced by 4 subcutaneous injections of 20 µg/kg body weight of cerulein (Sigma, St. Louis, MO, USA) at hourly intervals within 4 hours. Control animals received isotonic saline. Proanthocyanidin was administered orally at a dose of 100 mg/kg per rat 15 min before first cerulein injection. The dose of proanthocyanidin was previously shown as an effective anti-inflammatory dose [14]. Six hours after cerulein or saline injections, the animals were killed by decapitation. Trunk blood was collected for the assessment of amylase, lipase, TNF-α, IL-1β. In order to evaluate the presence of oxidant injury in the pancreas tissue, samples were taken and stored at -80 °C for the determination of malondialdehyde (MDA) and glutathione (GSH) levels, myeloperoxidase (MPO) and Na^+^-K^+^-ATPase activities. Formation of reactive oxygen species in the tissue samples was monitored by using chemiluminescence (CL) technique with luminol and lucigenin probes. For histological analysis, samples of the tissues were fixed in 10% (v/v) buffered formaldehyde and prepared for routine paraffin embedding. Tissue sections (6 µm) were stained with hematoxylin and eosin and examined under a light microscope (Olympus-BH-2). An experienced histologist who was unaware of the treatment conditions performed the histological assessments.

### Biochemical analysis

Plasma amylase and lipase levels were determined spectrophotometrically using an automated analyser (Olympus AU 600, Diamond Diagnostics, Holliston, MA) while tumor necrosis factor-alpha (TNF-α) and interleukin IL-1β were quantified according to the manufacturer’s instructions and guidelines using enzyme-linked immunosorbent assay (ELISA) kits (Biosource International, Nivelles, Belgium). These particular assay kits were selected because of their high degree of sensitivity, specificity, inter- and intra-assay precision and small amount of plasma sample required for conducting the assay.

### Chemiluminescence (CL) assay

To assess the contribution of reactive oxygen species in cerulein-induced pancreatic damage, luminol and lucigenin chemiluminescences were measured as indicators of radical formation. Measurements were made at room temperature using Junior LB 9509 luminometer (EG&G Berthold, Germany). Specimens were put into vials containing PBS-HEPES buffer (0.5 M PBS containing 20 mM HEPES, pH 7.2). ROS were quantified after addition of enhancers, lucigenin or luminal, for a final concentration of 0.2 mM. Luminol detects a group of reactive species, i.e. ^.^OH, H_2_O_2_, HOCl radicals, while lucigenin is selective for O^-^_2_ [15, 16]. Counts were obtained at 1 min intervals and the results were given as the area under curve (AUC) for a counting period of 5 min. Counts was corrected for wet tissue weight and expressed as relative light units (rlu/mg tissue) [17].

### Measurement of pancreatic malondialdehyde and glutathione levels

Tissue samples were homogenized with ice-cold 150 mM KCl for the determination of MDA and GSH levels. The MDA levels were assayed for the products of lipid peroxidation by monitoring thiobarbituric acid reactive substance formation as described previously [18]. Lipid peroxidation was expressed in terms of MDA equivalents using an extinction coefficient of 1.56 x 10^5^ M^–1^ cm ^–1^ and results were expressed as nmol MDA/g tissue. GSH measurements were performed using a modification of the Ellman procedure [19]. Briefly, after centrifuged at 1200 g for 10 min, 0.5 ml of supernatant was added to 2 ml of 0.3 mol/l Na_2_HPO_4_.2H_2_O solution. A 0.05 ml solution of 10 mM dithiobisnitrobenzoate (disolved in 1% sodium citrate) was added and the absorbance at 412 nm was measured immediately after mixing. GSH levels were calculated using an extinction coefficient of 1.36 x 10^4^ M^–1^ cm ^–1^. Results were expressed in µmol GSH/g tissue.

### Measurement of pancreatic myeloperoxidase activity

Myeloperoxidase (MPO) is an enzyme that is found predominantly in the azurophilic granules of polymorphonuclear leukocytes (PMN). Tissue MPO activity is frequently utilized to estimate tissue PMN accumulation in inflamed tissues and correlates significantly with the number of PMN determined histochemically in tissues [20]. MPO activity was measured in tissues in a procedure similar to that documented by Hillegass et al [21]. Tissue samples were homogenized in 50 mM potassium phosphate buffer (PB, pH 6.0), and centrifuged at 41400 g (10 min); pellets were suspended in 50 mM PB containing 0.5 % hexadecyltrimethylammonium bromide (HETAB). After three freeze and thaw cycles, with sonication between cycles, the samples were centrifuged at 41400 g for 10 min. Aliquots (0.3 ml) were added to 2.3 ml of reaction mixture containing 50 mM PB, o-dianisidine, and 20 mM H_2_O_2_ solution. One unit of enzyme activity was defined as the amount of MPO present that caused a change in absorbance measured at 460 nm for 3 min. MPO activity was expressed as U/g tissue.

### Measurement of Na^+^- K^+^ ATPase activity

Measurement of Na^+^, K^+^-ATPase activity is based on the measurement of inorganic phosphate that is formed from 3 mM disodium adenosine triphosphate added to the medium during the incubation period [22]. The medium was incubated in a 37 °C water bath for 5 min with a mixture of 100 mM NaCl, 5 mM KCl, 6 mM MgCl_2_, 0.1 mM EDTA, 30 mM Tris HCl (pH 7.4). Following the preincubation period, Na_2_ATP, at a final concentration of 3 mM, was added to each tube and incubated at 37 °C for 30 min. After the incubation, the tubes were placed in an ice bath, and the reaction was stopped. Subsequently, the level of inorganic phosphate was determined in a spectrophotometer (Shimadzu, Japan) at excitation wavelength of 690 nm. The specific activity of the enzyme was expressed as mmol Pi mg^-1^ protein h^-1^. The protein concentration of the supernatant was measured by the Lowry method [23].

### Histopathological evaluation of pancreatic damage

For light microscopic analysis, samples from pancreas were fixed in 10% buffered formalin for 48 hours, dehydrated in ascending alcohol series and embedded in paraffin wax. Approximately 5 mm thick sections were stained with hematoxylin-eosin (H&E) for general morphology. Histological assessments were made with a photomicroscope (Olympus BX 51, Tokyo) by an experienced histologist who was unaware of the experimental groups.

### Statistics

Statistical analysis was carried out using GraphPad Prism 3.0 (GraphPad Software, San Diego; CA; USA). All data were expressed as means ± SEM. Groups of data were compared with an analysis of variance (ANOVA) followed by Tukey’s multiple comparison tests. Values of p < 0.05 were regarded as significant.

## Results

As shown in Table 1, plasma amylase and lipase levels in the pancreatitis group were found to be significantly higher than those in control rats (p < 0.001). When proanthocyanidin was administered before cerulein injection, these elevations in serum amylase and lipase levels were significantly depressed (p < 0.01). Plasma levels of pro-inflammatory cytokines (TNF-α, IL-1β) in the pancreatitis group were significantly higher (p < 0.001) than that of the control group, while treatment of proanthocyanidin abolished these elevations significantly (p < 0.01).

**Table 1:** The plasma amylase, lipase, tumour necrosis factor-α (TNF-α) and interleukin-1β (IL-1β) levels in sham control group or cerulein-induced acute pancreatitis groups treated with either saline or proanthocyanidine (100 mg/kg). Each group consists of 6 animals.

	Control	Pancreatitis	
		Saline-treated	Proanthocyanidin-treated
Amylase (U/L)	646.03 ± 46.2	1202.82 ± 67.3 ***	666.80 ± 53.7 ^++^
Lipase (U/L)	114.92 ± 23.2	393.37 ± 38.5 ***	163.13 ± 26.7 ^++^
TNF-α (pg/ml)	5.37 ± 0.91	40.58 ± 8.54 ***	15.30 ± 2.76 ^++^
IL-1β (pg/ml)	9.52 ± 1.69	52.07 ± 5.99 ***	23.28 ± 4.99 ^++^

Data are the mean ± SEM of six animals. ***P < 0.001 compared to control group; *^++^P* < 0.01, compared with the saline-treated pancreatitis group.

**Figure 1:**
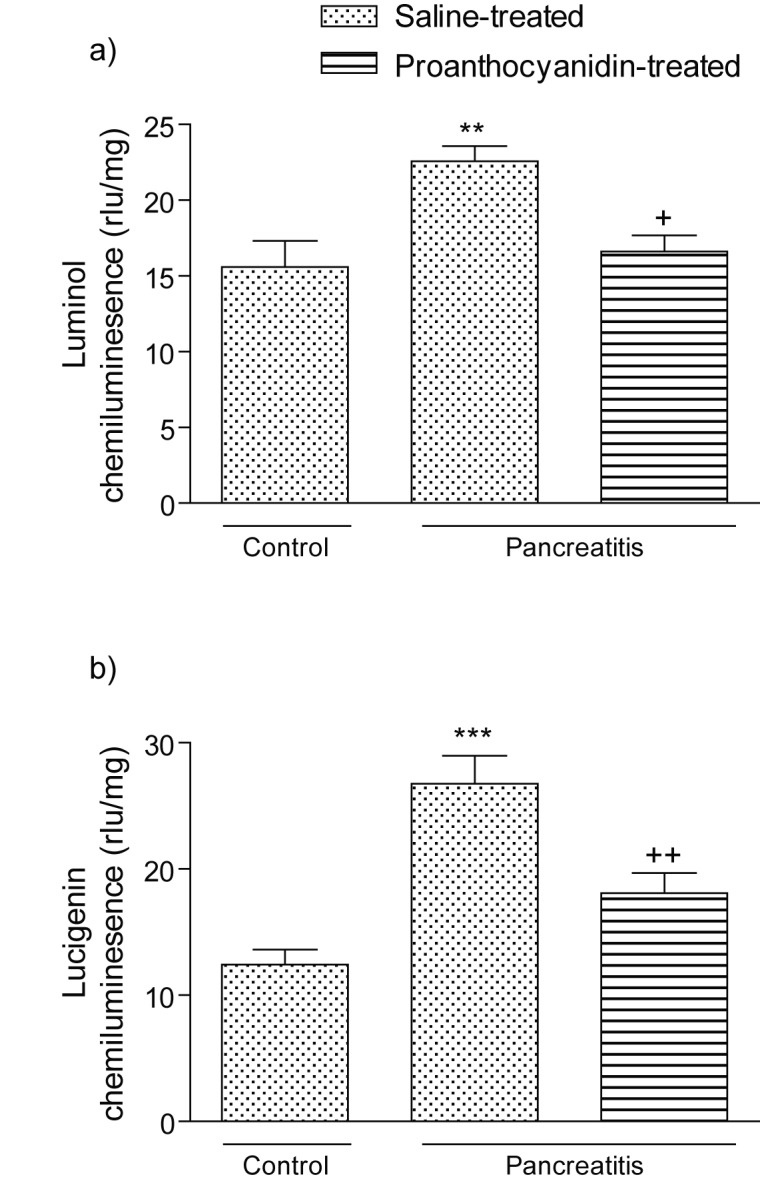
(a) Luminol and (b) lucigenin chemiluminescence (CL) in the pancreatic tissues of control, saline-treated pancreatitis and proanthocyanidin-treated pancreatitis groups. Each group consists of 6 animals. rlu: relative light units. ** p < 0.01, *** p < 0.001, compared to the control group. ^+^p < 0.05, ^++^p < 0.01, compared to saline-treated pancreatitis group.

Chemiluminescence levels in the pancreatic samples detected by both luminol and lucigenin probes showed significant increases in the vehicle-treated pancreatitis group as compared to the CL levels of the control group (p < 0.01-0.001; Fig. 1a and b). On the other hand, proanthocyanidin treatment in the pancreatitis group abolished the pancreatitis-induced increases in both lucigenin- and luminol-detected CL (p < 0.05-0.01).

**Figure 2:**
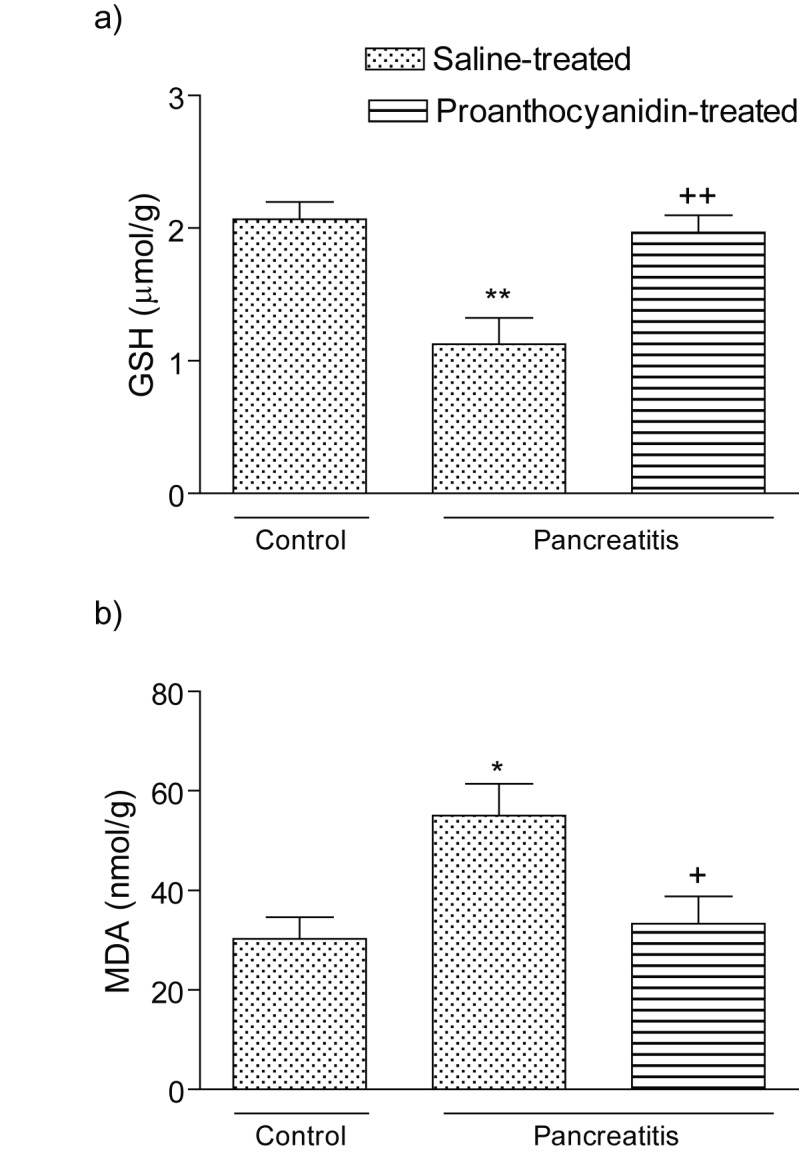
(a) Glutathione (GSH) level and (b) malondialdehyde (MDA) level in the pancreatic tissues of control, saline-treated pancreatitis and proanthocyanidin-treated pancreatitis groups. Each group consists of 6 animals. *p < 0.05, **p < 0.01, compared to the control group. ^+^p < 0.01, ^++^p < 0.001, compared to saline-treated pancreatitis group.

In accordance with these findings, levels of the major cellular antioxidant GSH in the vehicle-treated pancreatitis group was depleted (p < 0.01); however, in the proanthocyanidin treated pancreatitis group, depleted GSH stores were partially replenished with this antioxidant proanthocyanidin treatment (p < 0.01; Fig. 2 a). The MDA levels, measured as a major degradation product of lipid peroxidation in the pancreatic tissue, were found to be significantly higher in the pancreatitis group (p < 0.05) as compared to those of the control group, while treatment with proanthocyanidin abolished these elevations (p < 0.05; Fig. 2b).

**Figure 3:**
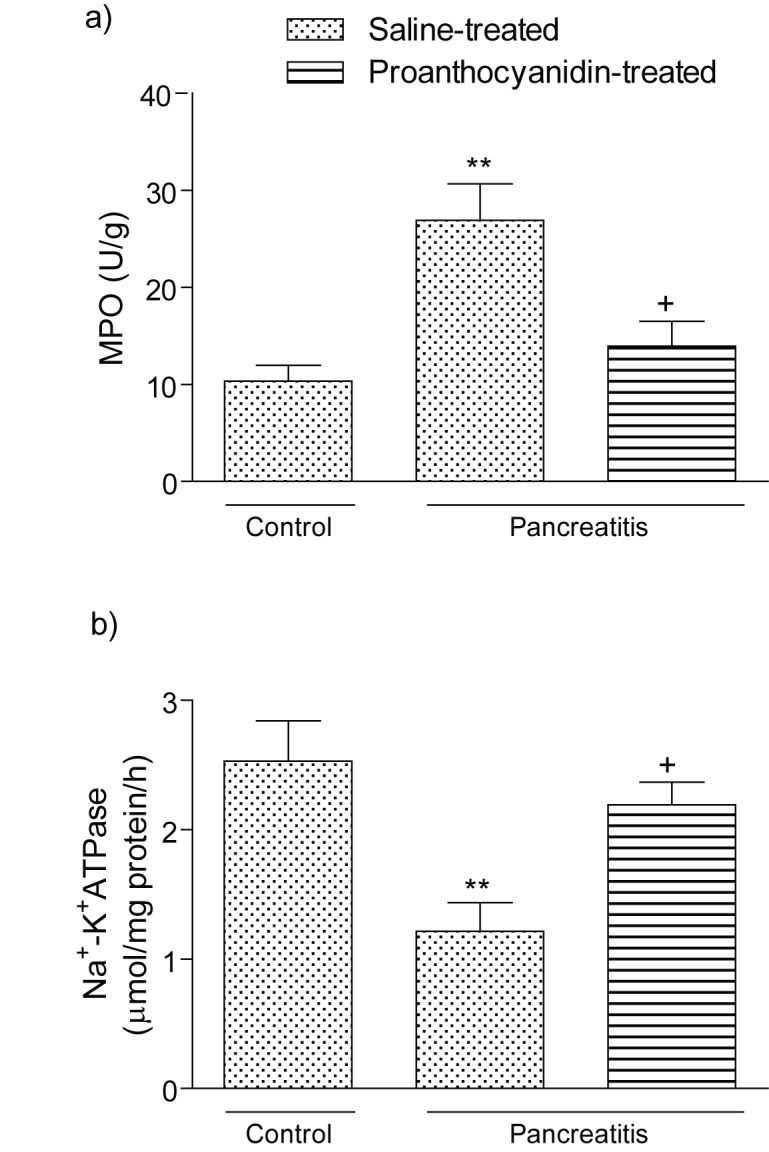
(a) Myeloperoxidase activity and (b) Na^+^-K^+^ ATPase activity in the pancreatic tissues of control, saline-treated pancreatitis and proanthocyanidin-treated pancreatitis groups. Each group consists of 6 animals. **p < 0.01, compared to the control group. ^+^p < 0.05, compared to saline-treated pancreatitis group.

Myeloperoxidase activity, which is accepted as an indicator of neutrophil infiltration, was significantly higher in the pancreatic tissue of the pancreatitis group treated with vehicle (p<0.01) than that of the control group (Fig. 3 a). On the other hand, proanthocyanidin treatment in the pancreatitis group significantly decreased pancreatic MPO level (p<0.05) back to the levels of the control group. The activity of Na^+^-K^+^ ATPase, indicating the functional transport capacity of the pancreatic cells, was found to be significantly decreased in the pancreatitis group as compared with control group (p < 0.01); however, proanthocyanidin treatment significantly reduced the cerulein-induced decrease in pancreatic Na^+^-K^+^ ATPase activity (p<0.05; Fig. 3b).

**Figure 4:**
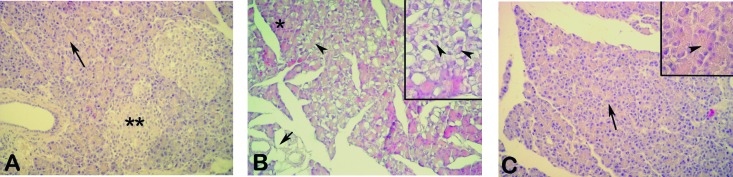
(A) Control group, regular acini (arrow) with Langerhans islets (**). (B) Cerulein treated group, severe degeneration of acini which shows thyroidization (arrowheads) with overall tissue vacuolization (arrow), note the partially intact acini (*), (C) Proanthocyanidin treated group, regenerated acini (arrow), loss of tissue vacuoles, cytoplasmic vacuolization (inset-arrowhead). HE, X200, insets X400.

Control pancreas tissues demonstrated a regular morphology with acinar structures and Langerhans islets (Fig. 4a).Cerulein treated tissues showed severe degeneration in acinar structures with thyroidization of acini and overall tissue vacuolization (Fig. 4b. In proanthocyanidin treated group, regeneration of acinar morphology and the loss of vacuolar structures were prominent (Fig. 4c), whereas the cytoplasm of acinar cells were still vacuolated.

## Discussion

One of the animal models of AP is that induced by supramaximal secretagogue stimulation by the trophic agent cerulein, a cholecystokinin analogue. Cerulein can stimulate the acinar cells to synthesize large amounts of digestive zymogens and pancreatic fluid, resulting in oedematous pancreatitis characterized by interstitial oedema, leukocyte infiltration and the vacuolization of acinar cells [4, 24, 25]. As assessed by both histological and biochemical parameters, the results of the present study demonstrated that cerulein caused oxidative injury of pancreatic tissues while proanthocyanidin treatment attenuates the severity of oxidative pancreatic damage along with concomitant reductions in the serum pro-inflammatory cytokines, suggesting that proanthocyanidin has a potent anti-inflammatory and anti-oxidant effect on the inflamed pancreatic tissue.

Although the pathophysiology is not fully understood, some of the early events of AP have been characterized by a dysregulation of the production and secretion of digestive enzymes, particularly, the inhibition of pancreatic secretion and an elevation in their serum levels, the death of acinar cells, and an infiltration of inflammatory cells into the pancreas [26-29]. Indeed, in our study, the amylase and lipase blood levels, indicators of the severity of acute pancreatitis [30], were increased following cerulein injection while, proanthocyanidin treatment reduced the levels of these enzymes.

The injured acinar cells release cytokines that attract neutrophils, activate platelets and the complement system. Indirectly, they act on the arachidonic acid cascade by increasing the production of thromboxane, which lowers tissue circulation by its potent platelet-aggregating and vasoconstricting effects, and by enhancing the production of leukotriene B_4_, which promotes the activation of leukocytes and discharges of lysosomal enzymes [31]. It is well known that severe form of acute pancreatitis is characterized by the development of systemic inflammatory response syndrome, which is reported to result in high mortality rates [32]. It has been suggested that pro-inflammatory cytokines, such as TNF-α and IL-1β are upregulated during pancreatitis [33]. These cytokines play important roles in the induction of PMN activation and infiltration and induce many local and systemic manifestations of acute pancreatitis. Recently, it has been shown that neutrophil accumulation and the cytokine content, including TNF-α and IL-1β, were increased in pancreatic injury [34]. In accordance with these findings, in the present study, the plasma levels of the pro-inflammatory cytokines TNF-α and IL-1β were significantly elevated due to cerulean injection while proanthocyanidin treatment reduced the levels of these inflammatory mediators and protected the pancreatic tissue against cerulein-induced oxidative injury.

Reactive oxygen metabolites (ROM) are involved in the development of tissue injury in pancreatitis, as well as in many inflammatory diseases [35]. In the current study, we investigated the free radical generation in the pancreatic tissue using chemiluminescence, a simple and reproducible technique for demonstrating the generation of oxidants in tissue. Chemiluminescence is a general assay for the production of reactive oxygen species, while cytochrome C reduction is a specific assay for superoxide anion. The luminol probe used in this technique detects H_2_O_2_, OH^-^, hypochlorite, peroxynitrite and lipid peroxyl radicals, while lucigenin is selective for superoxide radical [15-17]. Since increased CL values detected by both probes were significantly decreased with proanthocyanidin treatment, it seems likely that the protective effect of proanthocyanidin on the pancreatic tissue partly involves its direct antioxidant properties.

It is shown that acinar cells produce large amounts of ROS at early stage of AP in rats [36]. Highly reactive ROS directly attacks lipids, proteins in the biological membranes and cause their dysfunction [37]. Peroxidation of lipid membranes, disintegration of cytoskeleton and intracellular compartments by ROS might lead to the disturbances of digestive and liposomal enzymes transport within the acinar cell leading to this cell damage [30]. Degradation of polyunsaturated fatty acids in cell membranes by ROS results in the destruction of membranes and formation of thiobarbituric acid reactive substances, MDA or conjugated dienes as indicator of lipid peroxidation in the course of pancreatitis [38, 39]**.** In parallel to the CL results, the increased lipid peroxidation in the pancreatic tissue, as demonstrated by MDA assay, was also reversed with proanthocyanidin treatment, emphasizing the antioxidant action of proanthocyanidin on the deleterious consequences of ROMs in oxidative pancreatic injury. In accordance with our results, a previous report based on an experimental hepatoxicity and neurotoxicity model has demonstrated that proanthocyanidin treatment reduced lipid peroxidation and restored the transmembrane enzymes, thereby maintained the antioxidant status of the hepatic and brain cells [13].

Proanthocyanidin is an anti-inflammatory and antioxidant molecule, acting as an ROS scavenger, it promotes synthesis and accumulation of glutathione precursors [40]. Glutathione, the physiologically most important nonprotein antioxidant, is a major contributor to the intracellular reducing environment and acts as a scavenger of hydrogen peroxide and other peroxides [41]. In accordance with the previous reports, which have reported a marked and early depletion of GSH pancreatic tissue in different models of experimental pancreatitis [38, 42], we also showed GSH depletion in the pancreatic tissue. GSH plays a role in acinar stimulus-secretion coupling [43], in the maintenance of the cytoskeleton [44], and in appropriate protein folding in the endoplasmic reticulum [45]. Thus, depletion of intracellular GSH may contribute to impaired zymogen granule transport and to premature activation of pancreatic proenzymes [42]. Restoration of intracellular glutathione levels has been shown to ameliorate cerulein-induced pancreatitis in rat, suggesting that generation of reactive oxygen radicals and consequent depletion of glutathione play pivotal roles in the initiation of acute pancreatitis [39, 46]. Similarly in the present study, following cerulein injection, GSH was depleted; however, proanthocyanidin treatment restoring tissue GSH reduced the severity of pancreatitis. Thus, it is likely that proanthocyanidin increases the total amount of intracellular GSH and has an important role in the maintenance of this crucial antioxidant.

As we have also confirmed histologically in our pancreatitis model, commonly observed changes of pancreatic morphology during pancreatitis include various degrees of acinar cell damage, hemorrhage, and the recruitment of leukocytes into the damaged gland [47, 48]. MPO is an essential enzyme for normal neutrophil function, and when neutrophils are stimulated by various stimulants, MPO, as well as other tissue damaging substances, is released from the cells. Therefore, MPO is used as an index of tissue neutrophil infiltration [49]. In the present study, the cerulein-induced increase of MPO activity was significantly reduced by proanthocyanidin, suggesting that pancreatic oxidative damage involves the interaction of neutrophils, and the protective effect of proanthocyanidin on the pancreas depends on blockade of neutrophil infiltration. As activation of neutrophils might lead to the generation of reactive oxygen metabolites, the reduction in tissue neutrophil accumulation may also result in reduced lipid peroxidation and attenuated tissue injury. Proanthocyanidin treatment markedly reduced the MPO activity. Bomser et al [50] have shown that proanthocyanidin treatment reduced neutrophil infiltration. However, an exact explanation of the effects of proanthocyanidin on neutrophil activation remains unknown.

Proanthocyanidins, naturally occurring compounds widely available in fruits, vegetables, nuts, seeds, flowers and bark, are a group of polyphenolic bioflavonoids diverse in chemical structure, pharmacology and characteristics. Proanthocyanidins have been reported to exhibit a wide range of biological effects including antibacterial, antiviral, anti-inflammatory, antiallergic and vasodilatory actions [51-53]. Furthermore, proanthocyanidins have been reported to inhibit lipid peroxidation, platelet aggregation and capillary permeability and fragility and to modulate the activity of enzyme including cyclooxygenase and lipooxygenase [53]. Proanthocyanidins are believed to be nontoxic. If they are absorbed and biologically active *in vivo*, they may prevent free radical-mediated cytotoxicity and lipid peroxidation and protect low-density lipoproteins from oxidation [13].

Our results also indicate that cerulein impairs pancreatic Na^+^-K^+^-ATPase activity. The Na^+^-K^+^-ATPase, which is found exclusively in the pancreatic acini, plays a central role in pancreas electrolyte regulation and in the pathogenesis of pancreatic inflammation [54]. Since decreased Na^+^-K^+^-ATPase activity most likely reflects a diminished number of enzyme molecules due to a loss of Na^+^-K^+^-ATPase-containing mucosal cells [55], consequently, it also indicates severe mucosal inflammation and the loss of physiological function. Since proanthocyanidin treatment in the present study reversed cerulein-induced increment in MPO and reduction in Na^+^-K^+^-ATPase enzyme activity, it indicates that proanthocyanidin alleviates the pancreatic injury by preserving membrane structure.

During the past years, several substances have been tested, with varying degrees of success, in experimental models of acute pancreatitis as an attempt to modify the natural history of the disease by either blocking or neutralizing one or more inflammatory mediators which are involved in the pathophysiology of the disease [4, 39]. On the basis of our data, proanthocyanidin, by preventing free radical damaging cascades, oxidant radical release and through its membrane stabilizing effects, supports the maintenance of pancreatic integrity against acute inflammatory processes. Furthermore, proanthocyanidin augments the level of the main intracellular antioxidant glutathione which preserves the total antioxidant capacity in the pancreas. In conclusion, the results of the present study suggest that proanthocyanidin may have utility in treating acute pancreatititis.
